# A Laboratory Guide in Urinalysis and Toxicology

**Published:** 1889-09

**Authors:** 


**A Laboratory Guide in Urinalysis and Toxicology.** By R. A. WITTHAUS, A. M., M. D., Professor of Chemistry and Physics in the Medical Department, University of the City of New York; Professor of Chemistry and Toxicology in the Medical Department, University of Vermont, etc., etc. Second edition, cloth, pp. iv., 75. New York: William Wood & Company.

This little guide has reached a second edition, showing that it is appreciated by the working student. Its peculiar arrangement adds to its usefulness. The descriptions are clear and easily followed, and the blank leaves, for notes and the illustrations, all increase its practical value. We know of no better work of the kind, and advise students to follow it in their laboratory studies of the subjects it presents.

